# Association between bone mineral density and cardiovascular disease in older adults

**DOI:** 10.3389/fpubh.2023.1103403

**Published:** 2023-06-23

**Authors:** Yulu Yang, Yun Huang

**Affiliations:** Department of Geriatrics, Union Hospital, Tongji Medical College, Huazhong University of Science and Technology, Wuhan, Hubei, China

**Keywords:** bone mineral density, osteoporosis, cardiovascular disease, older adults, risk factor

## Abstract

**Background and aims:**

Cardiovascular disease and osteoporosis are common diseases in older adults with high morbidity. The study on the interaction between the two in pathogenic mechanisms has been paid much attention by the majority of researchers. This study aimed to explore the relationship between bone mineral density and cardiovascular disease in older adults.

**Methods:**

The primary data was downloaded from the National Health and Nutrition Examination Survey database of the United States. Multivariate logistic regression model, generalized additive model, and smooth curve fitting were used to explore the relationship between bone mineral density and cardiovascular events risk. When a curve relationship was found, a two-piecewise linear model was used to calculate the inflection point. In addition, subgroup analysis was also performed.

**Results:**

A total of 2097 subjects were included in this study. After adjusting for potential confounders, no significant association was found between lumbar bone mineral density and cardiovascular disease, while femur bone mineral density had a non-linear relationship with cardiovascular disease, with an inflection point of 0.741 gm/cm^2^. When bone mineral density was <0.741 gm/cm^2^, the risk of cardiovascular disease decreased speedily. Once bone mineral density exceeded this value, the risk of cardiovascular disease continued to decrease, but the trend became significantly slower. Compared with patients with normal bone mass, osteoporosis was associated with a 2.05-fold increased risk of cardiovascular disease (95% CI 1.68–5.52). There were no significant differences in interaction tests of all subgroups (*p* for interaction >0.05) except race.

**Conclusion:**

Our results indicated that bone mineral density was closely associated with the prevalence of cardiovascular disease in older adults over 60 years old, especially the femur bone mineral density was negatively non-linear associated with cardiovascular disease risk, with an inflection point of 0.741 gm/cm^2^.

## Introduction

Cardiovascular disease (CVD) ranks first among chronic non-communicable diseases in the world. It is characterized by high morbidity, disability, and mortality, seriously affecting the quality of life (1). According to the latest annual statistics of the American College of Cardiology, the overall prevalence of CVD in the adult population was 9.3% (26.1 million), and about 874,613 people died of CVD in 2019 (2). In Asia, a meta-analysis found that the probability of fatal cardiovascular events in a population free of CVD history at baseline was 3.68/per 1,000 person-years (3). With the acceleration of the population aging process, CVD will bring a greater social and economic burden, and how to effectively prevent and treat CVD is a huge challenge.

Osteoporosis is a multifactorial metabolic bone disease characterized by decreased bone mass and destruction of bone microstructure, resulting in increased bone fragility and fracture risk. The fundamental mechanism is the imbalance of bone homeostasis maintained by bone formation and bone destruction (4). The prevalence of osteoporosis in the world’s older adults was 21.7%, with the highest prevalence of 24.3% in Asian countries, followed by Europe (16.7%) and the United States (11.5%) (5). Dual-energy X-ray absorptiometry (DXA) is the most widely used diagnostic method of osteoporosis. The classification criteria for DXA measurement released by the World Health Organization were: normal bone mass (T score ≥ −1); osteopenia (T score > −2.5, and < −1); osteoporosis (T score ≤ −2.5); severe osteoporosis (T score ≤ −2.5, and accompanied with brittle fractures) (6, 7).

Recent animal experiments showed that the femur and lumbar bone mineral density (BMD) decreased by 6.9 and 3.5%, respectively, in the myocardial infarction mouse model established by artificial ligation of the left anterior descending artery (8). In addition, several populations’ clinical studies had reported a possible association between BMD and CVD occurrence. For example, Wiklund et al. found that lower BMD was associated with an increased risk of myocardial infarction in both men and women (9). Other epidemiological studies have reported an association between reduced BMD and higher morbidity and mortality in stroke (10) and heart failure (11).

In the present study, we conducted multivariate logistic regression and stratified analysis to explore the possible relationship between BMD and the risk of CVD in older adults over 60 years old. This study is expected to provide more guidance on early monitoring and clinical prevention.

## Methods

**Figure 1:**
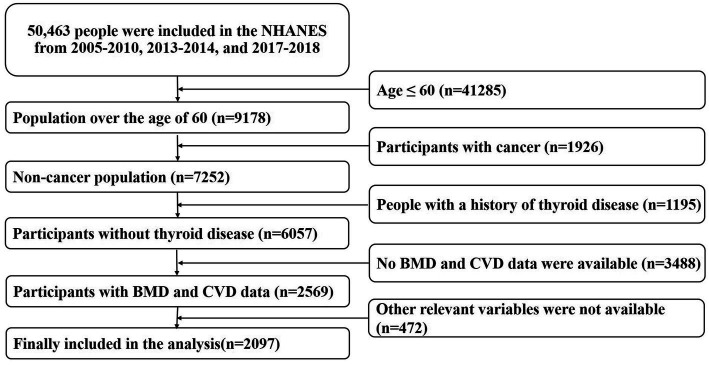
Screening criteria for the study population.

The raw data used in this study came from the National Health and Nutrition Examination Survey (NHANES) of the United States.^1^ NHANES is a nationwide cross-sectional study based on diverse levels of population. It integrates the demographics, dietary, examination, laboratory, questionnaire, and limited access data. This information will be used to evaluate the residents’ nutritional status and its association with disease prevention. In this study, we extracted the population data aged >60 years from 2005–2010, 2013–2014, and 2017–2018, to increase the sample size and improve statistical efficiency (BMD measurements in 2011–2012 and 2015–2016 were limited to people aged 8–59 years, so they were excluded). Detailed study design proposals were available on the NHANES website. In addition, we excluded patients with malignancy and thyroid disease (1926, 1,195, respectively). Subjects without BMD measurements (3,467 cases) and uncertain history of CVD (21 cases) were removed, too. 472 participants with missing other baseline data were also excluded and the remaining 2097 entered the final analysis. Detailed screening criteria for the study were provided in Figure 1. The NCHS Research Ethics Review Board (ERB) approved all agreements and each participant signed a written informed consent form.

CVD was defined as a range of self-reported diseases, including congestive heart failure, coronary heart disease, angina pectoris, and heart attack. These questions were described in the NHANES questionnaire dataset as MCQ160 b-e (has a doctor or other health professional ever told you that you had congestive heart failure, coronary heart disease, angina/angina, or heart attack?). If all the above-mentioned diseases were denied, the subject was considered to have no CVD. On the contrary, if one or more of the diseases were identified, the subject was considered to have developed CVD (12).

DXA is the most widely used method of BMD measurement in clinical practice, which has the advantages of fast speed, ease to use, and low radiation exposure. The lumbar and femur were scanned using the Hologic Discovery model A densitometer (Hologic, Inc., Bedford, Massachusetts). BMD measurements were performed by trained and certified radiologists, and those who were pregnant, had used contrast material in the last 7 days, and were overweight than required were excluded. Detailed information about BMD measurements and procedures can be found on the NHANES website.

Covariables include age, sex, race, systolic blood pressure, diastolic blood pressure, body mass index, waist circumference, hypertension, hyperlipidemia, diabetes mellitus, chronic kidney disease, liver disease, smoke, albumin, alanine aminotransferase, aspartate aminotransferase, blood urea nitrogen, creatinine, phosphorus, calcium, high-density lipoprotein cholesterol, total cholesterol, glycated hemoglobin, and Vitamin D. Relevant medical history can be found in the corresponding column in the questionnaire data. Hypertension was defined as a self-reported history of hypertension (BPQ020 Have you ever been told by a doctor or other health professional that you had hypertension, answered yes), or systolic blood pressure ≥ 140 mmHg, or diastolic blood pressure ≥ 90 mmHg. Hyperlipidemia was defined as a self-reported history of hyperlipidemia (BPQ080 Have you ever been told by a doctor or other health professional your blood cholesterol level was high, answered yes), or blood cholesterol concentration ≥ 5.7 mmol/L. Diabetes was defined as a self-reported history of diabetes (DIQ010 Have you ever been told by a doctor or other health professional that you had diabetes other than during pregnancy, answered yes), or glycated hemoglobin ≥6.5%. Chronic kidney disease was defined as a self-reported history of kidney disease (KIQ022 Have you ever been told by a doctor or other health professional that you had a weak or failing kidney, do not include kidney stones, bladder infections, or incontinence, answered yes), or creatinine ≥177 mmol/L. Liver disease was defined as a self-reported history of liver disease (MCQ160l has a doctor or other health professional ever told you that you had any kind of liver condition, answered yes), median liver stiffness ≥7.3 kPa or median controlled attenuated parameter≥240 dB/m measured by liver ultrasound transient elastography (13). Smoking status was determined by serum cotinine concentration (≥10 ng/mL was defined as a smoker, and < 10 ng/mL was a non-smoker) (14).

All statistical analyses were calculated using the R package, version 4.2.0,^2^ and EmpowerStats software.^3^ *p* < 0.05 indicated that the difference was statistically significant. Continuous variables were expressed as mean ± SD or median (interquartile range, IQR), while categorical variables were presented as percentages (%). Multivariable logistic regression models were performed to explore the relationship between BMD and CVD occurrence. After adjusting for confounding factors of CVD, a generalized additive model and smooth curve fitting were used to achieve visualization. When the nonlinear relationship was found, a two-piecewise linear regression model was used to analyze. Then, subgroup analyses were used to find the heterogeneity between different groups stratified by age, sex, race, body mass index, hypertension, hyperlipidemia, diabetes mellitus, chronic kidney disease, liver disease, and smoking status.

## Results

A total of 2097 people were included in this study. The description of baseline characteristics was shown in Table 1. 347 people suffered from CVD, with an incidence rate of 16.55%. The average age of the population was 68.92 ± 6.37, with males accounting for 51.88% and females for 48.12%. Non-Hispanic whites accounted for the highest proportion among different ethnic groups at 45.45%. Compared with the no CVD group, participants in the CVD group tended to be older, more male, more smokers, and have higher rates of hypertension, hyperlipidemia, diabetes, and chronic kidney disease. In addition, among different groups of diastolic blood pressure, body mass index, waist circumference, albumin, blood urea nitrogen, creatinine, calcium, high-density lipoprotein cholesterol, total cholesterol, glycosylated hemoglobin, vitamin D, and lumbar BMD, CVD occurrence was significantly different, whereas others were not.

**Table 1:** Baseline characteristics of patients (*n* = 2097).

Characteristic	Total	No CVD	CVD	*p*-value
*N*	2097	1750	347	
Age	68.92 ± 6.37	68.51 ± 6.25	70.99 ± 6.56	<0.001
*Sex*				<0.001
Male	1,088 (51.88%)	845 (48.29%)	243 (70.03%)	
Female	1,009 (48.12%)	905 (51.71%)	104 (29.97%)	
*Race*				<0.001
Mexican American	326 (15.55%)	287 (16.40%)	39 (11.24%)	
Other Hispanic	206 (9.82%)	178 (10.17%)	28 (8.07%)	
Non-Hispanic White	953 (45.45%)	758 (43.31%)	195 (56.20%)	
Non-Hispanic Black	425 (20.27%)	367 (20.97%)	58 (16.71%)	
Other	187 (8.92%)	160 (9.14%)	27 (7.78%)	
SBP, mmHg	134.51 ± 20.30	134.83 ± 20.21	132.91 ± 20.69	0.108
DBP, mmHg	68.73 ± 14.48	69.24 ± 14.66	66.13 ± 13.25	<0.001
BMI, kg/m^2^	27.94 ± 5.18	27.78 ± 5.12	28.76 ± 5.37	0.001
WC, cm	99.25 ± 13.44	98.42 ± 13.21	103.42 ± 13.84	<0.001
*Hypertension*				0.002
No	672 (32.05%)	585 (33.43%)	87 (25.07%)	
Yes	1,425 (67.95%)	1,165 (66.57%)	260 (74.93%)	
*Hyperlipidemia*				0.017
No	721 (34.38%)	621 (35.49%)	100 (28.82%)	
Yes	1,376 (65.62%)	1,129 (64.51%)	247 (71.18%)	
*DM*				<0.001
No	1,550 (73.92%)	1,335 (76.29%)	215 (61.96%)	
Yes	547 (26.08%)	415 (23.71%)	132 (38.04%)	
*CKD*				<0.001
No	2012 (95.95%)	1,697 (96.97%)	315 (90.78%)	
Yes	85 (4.05%)	53 (3.03%)	32 (9.22%)	
*Liver disease*				0.389
No	1775 (84.64%)	1,476 (84.34%)	299 (86.17%)	
Yes	322 (15.36%)	274 (15.66%)	48 (13.83%)	
*Smoke*				0.029
No	1740 (82.98%)	1,466 (83.77%)	274 (78.96%)	
Yes	357 (17.02%)	284 (16.23%)	73 (21.04%)	
ALT, U/L	20.00 (16.00–25.00)	20.00 (16.00–25.00)	20.00 (16.00–25.00)	0.251
Albumin, g/L	41.73 ± 3.02	41.79 ± 2.99	41.39 ± 3.14	0.026
AST, U/L	23.00 (20.00–27.00)	23.00 (20.00–27.00)	23.00 (20.00–27.00)	0.238
BUN, mmol/L	5.67 ± 2.34	5.53 ± 2.13	6.37 ± 3.11	<0.001
Creatinine, umol/L	81.33 (69.84–97.24)	79.56 (68.07–93.70)	90.17 (76.91–108.73)	<0.001
Phosphorus, mmol/L	1.20 ± 0.18	1.20 ± 0.18	1.19 ± 0.17	0.342
Calcium, mmol/L	2.36 ± 0.10	2.37 ± 0.10	2.35 ± 0.09	0.027
HDL-c, mmol/L	1.41 ± 0.43	1.43 ± 0.43	1.31 ± 0.39	<0.001
TC, mmol/L	5.12 ± 1.10	5.21 ± 1.08	4.66 ± 1.12	<0.001
HbA1c, %	6.06 ± 1.15	6.01 ± 1.12	6.29 ± 1.26	<0.001
Vitamin D, nmol/L	66.94 ± 27.28	67.37 ± 27.40	64.77 ± 26.56	0.105
Lumbar BMD, gm/cm^2^	0.99 ± 0.18	0.99 ± 0.18	1.03 ± 0.18	<0.001
Femur BMD, gm/cm^2^	0.91 ± 0.16	0.91 ± 0.16	0.91 ± 0.16	0.547

Mean ± SD or median (IQR) for continuous variables; *p*-value was calculated by the linear regression model.

% For categorical variables; the *p*-value was calculated by the chi-square test.

SBP, systolic blood pressure; DBP, diastolic blood pressure; BMI, body mass index; WC, waist circumference; DM, diabetes mellitus; CKD, chronic kidney disease; ALT, alanine aminotransferase; AST, aspartate aminotransferase; BUN, blood urea nitrogen; HDL-c, high-density lipoprotein cholesterol; TC, total cholesterol; HbA1c, glycated hemoglobin.

As shown in Table 2, three multivariate logistic regression models were constructed to investigate the association between CVD and BMD of the lumbar and femur respectively: crude model, without covariate adjustment; model 1, adjusting for age, sex, and race; model 2 was adjusted for all covariates. In this study, the BMD was quartered in order from small to large (Q1, Q2, Q3, Q4), and the risk of CVD was calculated, respectively. For lumbar BMD, a significant positive association with CVD was found only in the crude model (OR = 3.93, 95% CI 2.12–7.29), and no association was found in either model 1 or model 2 after adjustment. For femur BMD, there was no significant relationship between BMD and CVD risk in the crude model and model 1 (*p* = 0.5470, 0.0585, respectively). After adjusting for age, sex, race, and other relevant covariates (model 2), there was a significant negative association (OR = 0.18, 95% CI 0.06–0.50, *p* = 0.0010). That is, one unit increase in femur BMD was associated with an 82% reduction in CVD risk after adjusting for all relevant covariates. In addition, when BMD was transformed from a continuous variable into a categorical variable (Q1, Q2, Q3, Q4), the trend test was still significant (*p* for trend = 0.0402). At the same time, with the lowest femur BMD (Q1) as the reference group, the risk of CVD was decreased by 2, 21, and 34% in the Q2, Q3, and Q4 groups, respectively.

**Table 2:** Relationship between BMD and CVD.

	Crude model OR (95% CI)	*p*	Model 1 OR (95% CI)	*p*	Model 2 OR (95% CI)	*p*
*Lumbar BMD*	3.93 (2.12, 7.29)	<0.0001	1.63 (0.81, 3.31)	0.1730	0.97 (0.44, 2.13)	0.9451
Q1	Ref		Ref		Ref	
Q2	1.28 (0.89, 1.83)	0.1865	1.05 (0.72, 1.53)	0.8007	0.98 (0.66, 1.46)	0.9380
Q3	1.74 (1.23, 2.46)	0.0016	1.34 (0.92, 1.94)	0.1226	1.16 (0.78, 1.72)	0.4746
Q4	2.07 (1.48, 2.91)	<0.0001	1.34 (0.92, 1.95)	0.1331	1.06 (0.70, 1.60)	0.7870
*p* for trend	<0.0001		0.0697		0.6490	
*Femur BMD*	1.24 (0.61, 2.51)	0.5470	0.43 (0.18, 1.03)	0.0585	0.18 (0.06, 0.50)	0.0010
Q1	Ref		Ref		Ref	
Q2	1.18 (0.85, 1.65)	0.3206	0.99 (0.69, 1.41)	0.9461	0.98 (0.67, 1.42)	0.8964
Q3	1.20 (0.86, 1.67)	0.2883	0.92 (0.63, 1.33)	0.6402	0.79 (0.53, 1.19)	0.2561
Q4	1.27 (0.91, 1.76)	0.1622	0.83 (0.56, 1.23)	0.3574	0.66 (0.43, 1.04)	0.0712
*p* for trend	0.1818		0.3061		0.0402	

Crude model adjusted for none.

Model 1 adjusted for age, sex, and race.

Model 2 adjusted for age, sex, race, SBP, DBP, BMI, WC, hypertension, hyperlipidemia, DM, CKD, liver disease, smoke, ALT, Albumin, AST, BUN, Creatinine, Phosphorus, Calcium, HDL-c, TC, HbA1c, Vitamin D.

Lumbar BMD: Q1 ≤ 0.863gm/cm^2^; Q2 0.864–0.987gm/cm^2^; Q3 0.988–1.103gm/cm^2^; Q4 ≥ 1.104gm/cm^2^.

Femur BMD: Q1 ≤ 0.791gm/cm^2^; Q2 0.792–0.902gm/cm^2^; Q3 0.903–1.012gm/cm^2^; Q4 ≥ 1.013gm/cm^2^.

The study population was further divided into normal bone mass, osteopenia, and osteoporosis by comparing with the peak BMD of healthy adults of the same sex, to analyze the relationship between osteoporosis and the risk of CVD (15) (Table 3). The study found that osteoporosis was significantly associated with an increased risk of CVD in model 1 and mode 2 (OR = 2.06, 3.05, 95% CI 1.20–3.52, 1.68–5.52, respectively). After adjusting for all potential covariates (model 2), osteoporosis was associated with a 2.05-fold increased risk of CVD compared with the normal group. However, no significant association was found between osteopenia and the risk of CVD.

**Table 3:** Relationship between osteoporosis and CVD.

	CVD	No CVD	Crude model	Model 1	Model 2
			OR (95%CI)	OR (95%CI)	OR (95%CI)
Normal	207	1,074	Ref	Ref	Ref
Osteopenia	117	600	1.01 (0.79, 1.30)	1.06 (0.81, 1.37)	1.22 (0.91, 1.64)
Osteoporosis	23	76	1.57 (0.96, 2.56)	2.06 (1.20, 3.52)	3.05 (1.68, 5.52)
*P* for trend			0.2433	0.0746	0.0034

Crude model adjusted for none.

Model 1 adjusted for age, sex, and race.

Model 2 adjusted for age, sex, race, SBP, DBP, BMI, WC, hypertension, hyperlipidemia, DM, CKD, liver disease, smoke, ALT, Albumin, AST, BUN, Creatinine, Phosphorus, Calcium, HDL-c, TC, HbA1c, Vitamin D.

**Figure 2:**
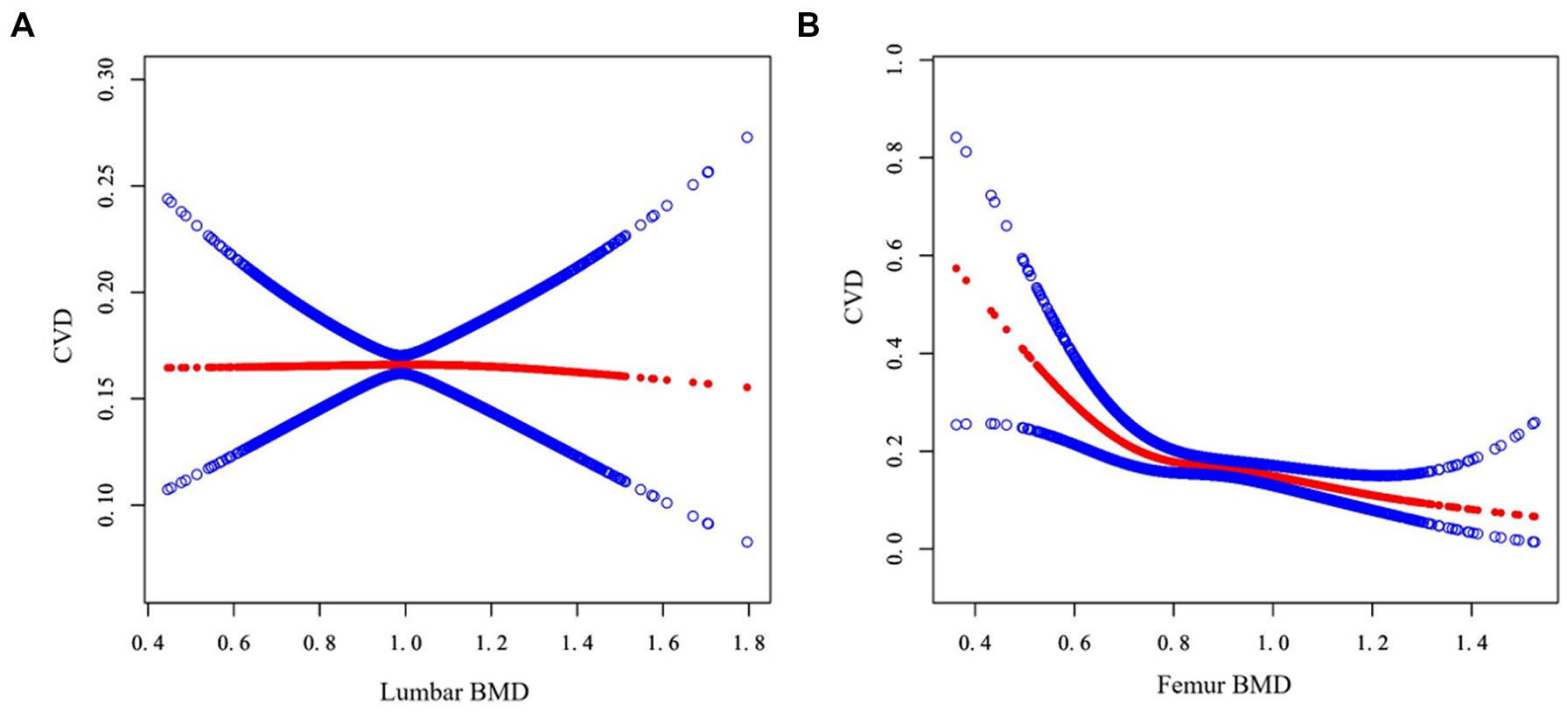
Association between BMD and CVD. The abscissa represents the BMD of the lumbar **(A)** and femur **(B)** respectively, and the ordinate represents the risk of developing CVD. The blue area shows the 95% confidence interval.

To further understand the true relationship between BMD and the risk of CVD, the present study also tried to assess the association using a generalized additive model and smooth curve fitting. Figure 2 showed the correlation trend between lumbar BMD and femur BMD and CVD risk, respectively. As can be seen, femur BMD appeared to be curvedly related to CVD risk, while lumbar BMD was not. This non-linear relationship would be further verified next.

**Figure 3:**
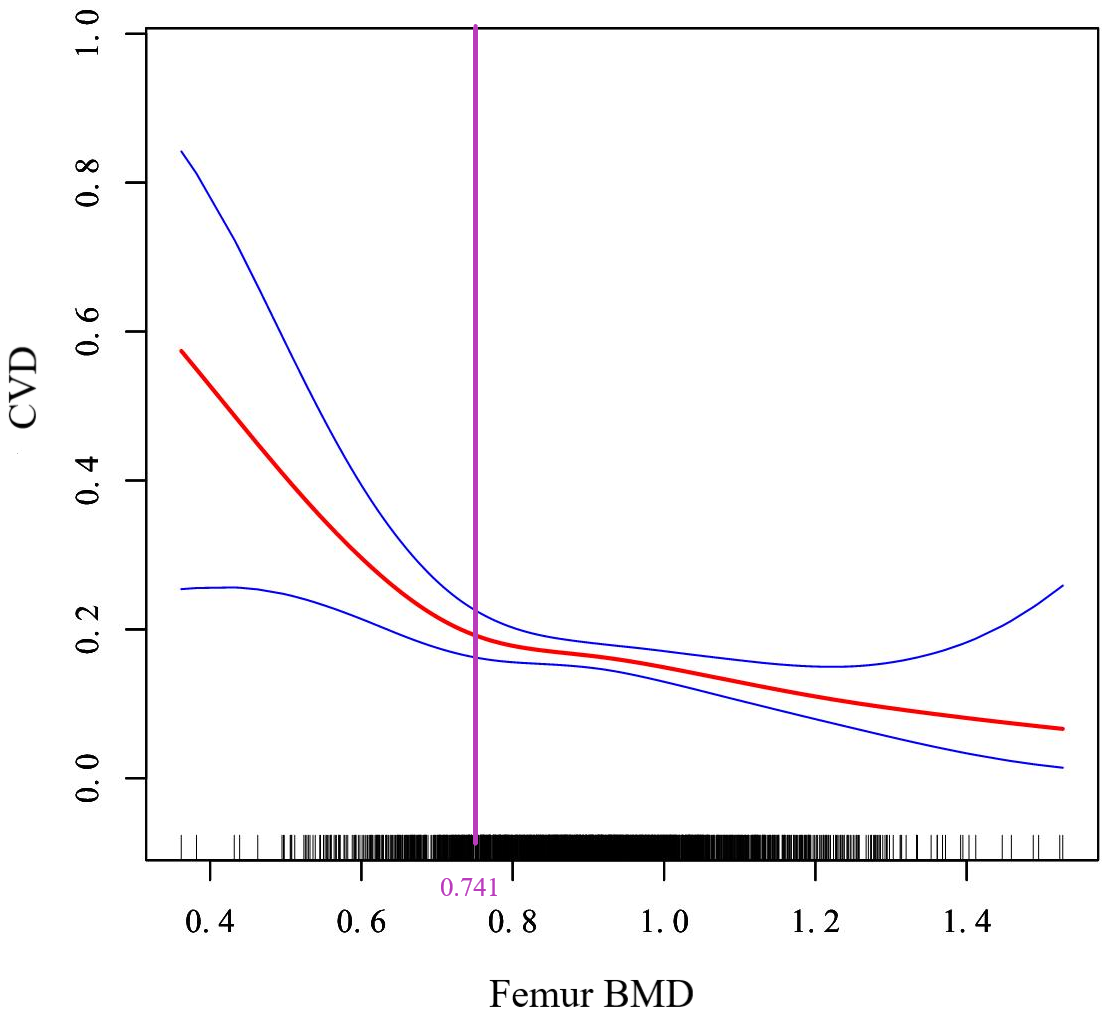
Curve association between femur BMD and CVD. The solid red line in the middle represents the trend of CVD risk as the femur BMD increases, and the dashed blue lines on either side represent the 95% confidence interval. The purple vertical line is the calculated inflection point.

Next, a two-piecewise linear regression model was used to analyze the threshold effect. Since the log-likelihood ratio test was *p* < 0.05, we believed that there was a curved relationship between femur BMD and the risk of CVD, and the inflection point was calculated to be 0.741gm/cm^2^ (Table 4). As shown in Figure 3, when BMD <0.741gm/cm^2^, the risk of CVD decreased rapidly with the increase of femur BMD. When the femur BMD was >0.741gm/cm^2^, the risk of CVD was further reduced, but the rate of decline was slightly slowed (OR = 0.003, 0.319, respectively).

**Table 4:** Threshold effect analysis of femur BMD and CVD using two-piecewise linear regression.

	Effect size (OR)	95% CI	*p*-value
*Model 1*			
Fitting by the standard linear model	0.180	(0.065, 0.502)	0.0010
*Model 2*			
Fitting by the two-piecewise linear model			
Inflection point	0.741		
< 0.741 gm/cm^2^	0.003	(0.000, 0.094)	0.0008
> 0.741 gm/cm^2^	0.319	(0.105, 0.974)	0.0448
Log-likelihood ratio	0.018		

Subsequently, we conducted a subgroup analysis stratified by age, sex, race, body mass index, hypertension, hyperlipidemia, diabetes, chronic kidney disease, liver disease, and smoke, and the results were shown in Table 5. There were no significant differences in the interaction test (*P* for interaction >0.05) except race, indicating that the relationship between femur BMD and CVD was not different between age, gender, and comorbidities.

**Table 5:** Effect size of femur BMD on CVD in subgroups.

	OR	95% CI	*p* for Interaction
*Sex*			0.2377
Male	0.17	(0.05,0.59)	
Female	0.15	(0.02,0.97)	
*Age*			0.3111
<70	0.26	(0.06,1.12)	
≥70	0.11	(0.02,0.48)	
*Race*			0.0480
Mexican American	3.16	(0.20, 50.12)	
Other Hispanic	0.02	(0.00, 3.02)	
Non-Hispanic White	0.17	(0.04, 0.74)	
Non-Hispanic Black	0.03	(0.00, 0.35)	
Other	0.12	(0.00, 15.69)	
*BMI*			0.5598
<25	0.08	(0.01,0.64)	
≥25, <30	0.21	(0.04,1.10)	
≥30	0.30	(0.05,1.78)	
*Hypertension*			0.7886
No	0.23	(0.03,2.14)	
Yes	0.17	(0.05,0.57)	
*Hyperlipidemia*			0.8952
No	0.15	(0.02,1.08)	
Yes	0.18	(0.05,0.61)	
*DM*			0.7395
No	0.20	(0.05,0.74)	
Yes	0.11	(0.02,0.62)	
*CKD*			0.8349
No	0.17	(0.06,0.51)	
Yes	0.27	(0.001,86.12)	
*Liver disease*			0.7677
No	0.16	(0.05,0.48)	
Yes	0.18	(0.01,3.93)	
*Smoke*			0.3372
No	0.26	(0.08,0.82)	
Yes	0.03	(0.00,0.42)	

## Discussion

The relationship between BMD and CVD disease is interesting, and this study does yield some very valuable findings. Firstly, this was a cross-sectional study of 2097 people over 60 years old (mean age 68.92, 51.88% male). After adjusting for all potential confounders, no significant correlation was found between lumbar BMD and CVD, while there was a non-linear relationship between femur BMD and the risk of CVD, with an inflection point of 0.741gm/cm^2^. Further, osteoporosis was associated with a significantly increased risk of CVD compared with those with normal bone mass (OR = 3.05, 95% CI 1.68–5.52). In addition, there were no significant differences among different groups of age, sex, body mass index, hypertension, hyperlipidemia, diabetes, chronic kidney disease, liver disease, and smoking. The results of this study provided powerful evidence support for the risk factors of CVD.

Recent studies have also reported the relationship between BMD and the risk of CVD, which is consistent with the conclusion of this study. Iseri and colleagues found that patients with higher Framingham cardiovascular risk scores tended to have lower head BMD (*p* < 0.001) (16). BMD was also significantly reduced in patients with abnormal myocardial perfusion or impaired left ventricular ejection fraction (*p* < 0.05) (17). Coronary artery calcification is a hallmark pathological change of coronary heart disease. Wiegandt found a negative correlation between BMD and coronary artery calcification (18). Of course, apart from BMD, higher cortical bone status and bone strength were associated with a lower risk of major cardiovascular adverse events after adjusting for confounders (19). In addition, several studies have reported the relationship between BMD and CVD outcomes. For example, a prospective cohort study from the UK Biobank found that osteoporosis was strongly associated with cardiovascular mortality in men (20). A cohort study of chronic heart failure in Japan found that patients with osteoporosis had a significantly increased incidence of adverse events, such as hospitalization or death (HR = 2.40, 95% CI 1.36–4.22) (21). Bisphosphonate is the first-line drug for the treatment of osteoporosis. A recent survey in China found that bisphosphonate significantly reduced the risk of all-cause mortality in patients with acute coronary syndrome or ischemic stroke (22).

CVD is the first major killer threatening human health. The research on its risk factors has been deeply concerned by both researchers and clinicians. According to relevant literature in recent years, BMD itself is also closely related to the main risk factors of CVD. For example, in a cross-sectional study of Japanese women, patients with essential hypertension had significantly lower BMD compared with the control group. Data also showed that BMD was particularly closely related to systolic blood pressure than diastolic blood pressure (23). In a survey of the community population in western China, the relationship between total cholesterol, low-density lipoprotein cholesterol, and high-density lipoprotein cholesterol and BMD of postmenopausal women showed a U-shaped curve. That is, on the left side of the inflection point, BMD was negatively correlated with these lipid indexes, while on the right side, they were positively correlated (24). What’s more, diabetes status was also related to BMD. Z score of heel BMD in premenopausal women with type 1 diabetes was significantly lower than that in the control group, and serum bone resorption markers, such as tartrate-resistant acid phosphatase-5b, were significantly higher than the control (25). While the respiratory risks of smoking are well known, the effects on BMD are not quite clear. An epidemiological study in South Korea found that tobacco exposure resulted in a significant decrease in BMD (*p* < 0.001), while a healthy lifestyle, such as avoiding sedentary jobs and increasing physical activities, was positively correlated with BMD (26). All of these have demonstrated the internal relationship between BMD and the risk of CVD, which are expected to guide primary prevention and clinical treatment.

In this study, the types of CVD included congestive heart failure and coronary heart disease. On the one hand, they are the most common diseases among the older adults, on the other hand, they are also the main causes of disability and death. Heart failure is a common manifestation of end-stage CVD, including rheumatic heart disease, hypertensive heart disease, myocardial disease, and ischemic heart disease (27). Here are several studies that have looked at BMD and heart failure. Fohtung and colleagues found that lower total hip BMD was associated with a higher risk of heart failure in a population over 65 years of age, and there were differences by gender and ethnicity (28). A large European Norfork epidemiological study found that a 1sd increase in BMD was associated with a 23% reduction in the risk of heart failure (29). A recent meta-analysis also showed that compared with healthy individuals, patients with chronic heart failure had more bone loss and lower total BMD, and further stratified analysis observed similar effects in the femoral neck, arm, leg, and trunk (30). Coronary heart disease (CHD) ranks first among CVD in the older adults and is mediated by atherosclerotic plaque. Angina pectoris and myocardial infarction, as the main clinical types of CHD seriously harm the health of the older adults. To date, few studies have shown a direct link between angina pectoris alone and BMD. However, studies on BMD and myocardial infarction are not scarce. For example, a cross-sectional study from NHANES III in the United States found that low BMD was associated with an increased incidence of myocardial infarction in older adults aged 50–79 years (OR = 1.28, 95% CI 1.01–1.63) (31). However, there are also some contradictory conclusions. For example, Pittman found that increased BMD through the use of anti-resorptive drugs increased the risk of myocardial infarction (HR = 1.38) (32). Though it was not ruled out that the above adverse effects were caused by drug side effects, prospective scientific trials are still needed to further analyze and verify these findings in the future.

It should be noted that no gender difference between BMD and the risk of CVD was observed in the sex-specific stratified analysis (Table 5). However, according to a large number of epidemiological surveys, women were more prone to suffer from osteoporosis than men, especially postmenopausal women (33). Estrogen deficiency was considered to be an important cause, so the guidelines have always recommended estrogen or estrogen receptor modulators for the prevention and treatment of perimenopausal or postmenopausal women (34). The mechanism of estrogen in osteoporosis was quite complex. On the one hand, estrogen stimulated bone formation by acting directly on osteoblasts; On the other hand, estrogen also inhibited osteoclast formation by regulating some cytokines and growth factors. In addition, estrogen also regulated bone metabolism by regulating the expression of various hormones, such as promoting calcitonin secretion and enhancing liver 25-hydroxylase and renal 1α -hydroxylase activities (35). Therefore, in the actual clinical personalized decision-making, the gender difference between patients and menstrual status is also an aspect that doctors must consider and pay attention to.

CVD and osteoporosis are often comorbidities in the older adults. Whether they simply coexist or interact with each other in pathogenesis is still controversial (36). Although the concept of the bone-vascular axis has been proposed for a long time (37, 38) and a growing body of evidence links abnormal BMD or bone metabolism with the risk of CVD, the specific cellular and molecular mechanisms remain unclear. Based on the latest research results from recent years, it may be related to the following aspects: First, there were common risk factors, common genetic and pathological mechanisms, as well as the causal association between osteoporosis and CVD, so they interacted and influenced each other (39). Second, the presence of vascular calcification might be the most important factor explaining the association between them. Vascular calcification was an active and complex process, especially with age, calcium was gradually lost from the bones and deposited in the cardiovascular system, setting off a host of diseases (40). To be specific, with bone loss, vascular smooth muscle cells transformed into osteoblast phenotype through increasing the level of matrix metalloproteinase-2 and transactivating the RunX promoter (41, 42), which led to vascular calcification and increased hardness, affecting the hemodynamics of the cardiovascular system. What’s more, another possible factor was low levels of inflammation, which played a catalytic role in the reduction of BMD (43) and was a crucial role in the pathogenesis of atherosclerotic vascular disease (44). There are several clinical trials targeting inflammation are currently under investigation. Last but not least, people with poor bone health tended to be weaker and less physically active, especially those with combined fractures, and prolonged bedridden conditions significantly increased the risk of CVD (45).

Based on the results of this study, it can be roughly speculated that DXA examination or targeted prevention strategies, such as increased sun exposure, appropriate physical exercise, and calcium or vitamin D supplement, can be considered for patients with CVD. Meanwhile, for patients with osteoporosis or those at high risk of fracture, active anti-osteoporosis drug therapy can increase BMD and improve bone quality and reduce cardiovascular complications to a certain extent. Of course, large randomized controlled trials are needed before BMD measures are widely used to guide the treatment of patients with CVD.

Admittedly, there are some limitations. Firstly, this was a cross-sectional study based on the target population, making it difficult to determine the exact causal relationship between BMD and CVD. Secondly, all the samples used for analysis in this study were from the NHANES database. Although these samples represented the American population well, further research with multi-center data from other countries and regions is still needed. Thirdly, the study excluded patients with malignant tumors and thyroid diseases, which were common causes of secondary osteoporosis, so it was not possible to evaluate the applicability to these populations. Fourthly, the prevalence of CVD in this study was calculated according to the patient’s self-reported medical history, which inevitably resulted in recall bias and reporting bias. In addition, due to the limitation of the database, this study only included heart failure and coronary heart disease, so it was not possible to assess the effect of BMD on other CVD. Finally, there were also potential variables not included that may cause bias, such as markers of serum bone turnover, inflammatory parameters, and dietary intake. In recent years, with the standardization of testing procedures, bone turnover markers are increasingly used in the routine management of osteoporosis, especially in pharmacodynamic evaluation (46, 47). International guidelines also recommend its measurement as an alternative to continuous BMD testing in mainstream clinical practice. However, it is still a long way to go to conduct a comprehensive evaluation with multi-center and large sample studies in the future. It should be noted that the normal range of reference values for different populations is also a factor worth considering.

## Conclusion

In conclusion, there was a negative non-linear relationship between the level of femur BMD and the prevalence of CVD in people over 60 years of age, with an inflection point of 0.741gm/cm^2^. No significant differences were found between age, gender, and comorbidities subgroups. Bone loss can be considered as a new risk factor for CVD, and future studies need to make a comprehensive assessment combining dietary and serum indicators. Therefore, efforts to prevent osteoporosis are of great importance, as this may indirectly reduce the prevalence of CVD, the world’s biggest killer of humans.

## Data availability statement

The raw data supporting the conclusions of this article will be made available by the authors, without undue reservation.

## Ethics statement

The studies involving human participants were reviewed and approved by Protocol #2018-01 NCHS Ethics Review Board. The patients/participants provided their written informed consent to participate in this study.

## Author contributions

YY: data curation, formal analysis, visualization, and writing—original draft. YH: conceptualization, project administration, supervision, validation, writing—review and editing, and supervision. All authors contributed to the article and approved the submitted version.

## Conflict of interest

The authors declare that the research was conducted in the absence of any commercial or financial relationships that could be construed as a potential conflict of interest.

## Publisher’s note

All claims expressed in this article are solely those of the authors and do not necessarily represent those of their affiliated organizations, or those of the publisher, the editors and the reviewers. Any product that may be evaluated in this article, or claim that may be made by its manufacturer, is not guaranteed or endorsed by the publisher.

## Footnotes

^1^ https://www.cdc.gov/nchs/nhanes/index.htm

^2^ http://www.r-project.org

^3^ http://empowerstats.com
